# Number of Chairs in Medical Schools

**Published:** 1849-06

**Authors:** 


### NUMBER OF CHAIRS IN MEDICAL SCHOOLS.

While in this country the tendency of the profession is to increase the number of chairs in medical schools, in Paris a disposition to the reverse has recently manifested itself. It has been proposed by the Committee of Education to suppress as useless or subordinate, the chair of clinical medicine; the chair of clinical surgery; the chair of medical pathology; the chair of surgical pathology; and the chair of hygiene, which will be reunited to that of physics.

These retrenchments will affect five of the most illustrious members of the faculty: MM. Fouquier, Roux, Dumeril, Marjolin, and Royer Collard. The first four having completed the term of service required by law, thirty years, will receive the full amount of the pension awarded to those whose period of enlistment has expired. M. Royer Collard, although he has not served the requisite length of time, will, because of his physical infirmities, also receive a full pension.

